# Emerging small-molecule treatments for multiple sclerosis: focus on B cells

**DOI:** 10.12688/f1000research.16495.1

**Published:** 2019-03-01

**Authors:** Aaron Gregson, Kaitlyn Thompson, Stella E Tsirka, David L Selwood

**Affiliations:** 1 The Wolfson Institute for Biomedical Research, University College London, Gower Street, London, WC1E 6BT, UK; 2 Department of Pharmacological Sciences, Stony Brook University, Stony Brook, New York, 11794, USA

**Keywords:** Multiple sclerosis, MS, B cells, small molecule drugs, sustainable healthcare

## Abstract

Multiple sclerosis (MS) is a major cause of disability in young adults. Following an unknown trigger (or triggers), the immune system attacks the myelin sheath surrounding axons, leading to progressive nerve cell death. Antibodies and small-molecule drugs directed against B cells have demonstrated good efficacy in slowing progression of the disease. This review focusses on small-molecule drugs that can affect B-cell biology and may have utility in disease management. The risk genes for MS are examined from the drug target perspective. Existing small-molecule therapies for MS with B-cell actions together with new drugs in development are described. The potential for experimental molecules with B-cell effects is also considered. Small molecules can have diverse actions on B cells and be cytotoxic, anti-inflammatory and anti-viral. The current B cell–directed therapies often kill B-cell subsets, which can be effective but lead to side effects and toxicity. A deeper understanding of B-cell biology and the effect on MS disease should lead to new drugs with better selectivity, efficacy, and an improved safety profile. Small-molecule drugs, once the patent term has expired, provide a uniquely sustainable form of healthcare.

## Introduction

Multiple sclerosis (MS) affects over two million people worldwide (National Multiple Sclerosis Society, https://www.nationalmssociety.org/). The disease is characterised by an initial autoimmune-driven, inflammatory phase followed by immune-mediated attack on the myelin surrounding nerve axons. Focal cortical lesions may develop in distinct locations. Eventually, the ensuing damage results in progressive nerve loss and increasing disability evident as disrupted motor function, visual disturbances, and bladder problems ^1^. As disease onset is often reported in young adults, its course can affect people for most of their adult lives. MS is considered to present in several forms: relapsing-remitting MS (RRMS), secondary progressive MS (SPMS), primary progressive MS (PPMS) and progressive-relapsing MS (PRMS). Diagnosis of the disease is complex; there is no single test for MS, and multiple criteria, including magnetic resonance imaging (MRI) scans are used ^2^.

**Figure 1.**
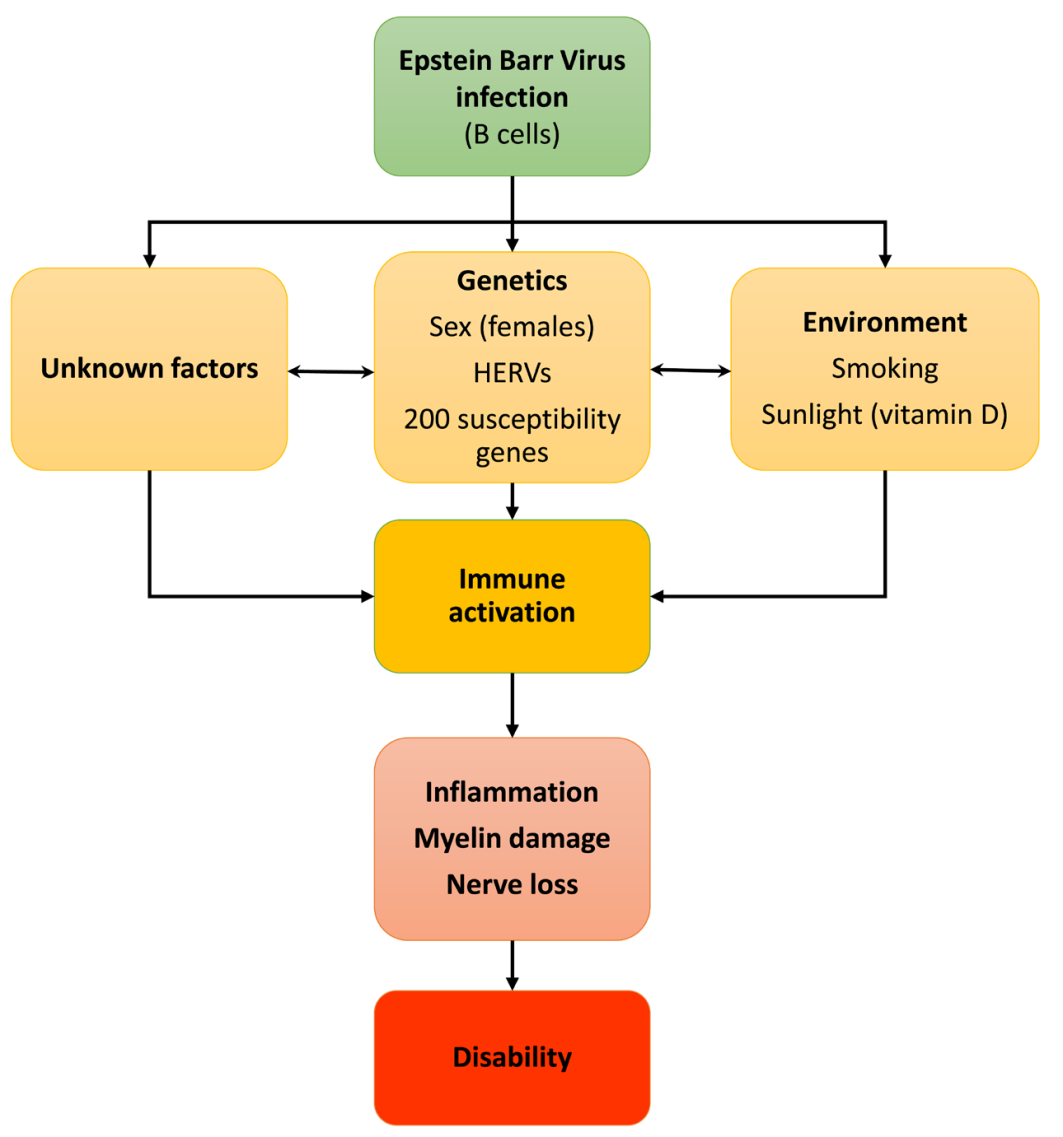
Causes and progression of multiple sclerosis (MS). Several studies now indicate that Epstein–Barr virus infection is necessary (but not causal) for MS to develop. Genetic factors may explain 50% of MS susceptibility whereas environmental factors together with unknowns may combine to trigger immune activation and the subsequent destruction of myelin and oligodendrocytes. This eventually leads to axonal damage and nerve cell death resulting in disability. HERV, human endogenous retrovirus.

The causes of the disease are multifactorial, with infectious, genetic and environmental factors, such as lack of sunlight (through vitamin D), playing a role ( Figure 1) ^3^. Large genome-wide association studies (GWASs) have gradually allowed the risk genes associated with MS to be elucidated. Up to now, genetics alone has not proven useful in diagnosis ^4^, but the identified risk genes have been informative about the mechanism and contributors to the disease and may be of aid in predicting severity ^5^. Among the more than 200 risk genes, most with a link to immune function, identified as susceptibility factors, the strongest associations are with the *HLA-DRB1* (human leucocyte antigen) locus in the major histocompatibility complex (MHC) ^4^.

Although multiple types of immune cells have been implicated in the pathology of MS [4], the role of B cells has recently come to the fore ^6^; notable clinical successes for agents which target B cells, such as CD20-targeted antibodies, rituximab, ocrelizumab and ofatumumab, are reported. In addition, an analysis of agents used to treat MS indicated that activity against a specific subset of B cells, the CD19 ^+^CD27 ^+^ memory B cells, correlated with clinical efficacy ^7– 9^.

Despite this strong driver to develop new B cell–directed therapies, the current most popular animal model used to study MS-like pathologies, particularly inflammation and neurodegeneration—experimental autoimmune encephalomyelitis (EAE) in mice—does not allow an assessment of a causative role for B-cell involvement, complicating further development ^10^. A review of animal models unsurprisingly points to primate models, such as the marmoset, as the most representative of the human disease ^11^.

The recent focus on how B cells contribute to MS pathology also renews interest in the role of Epstein–Barr virus (EBV) infection in the aetiology of the disease. EBV is present in a high percentage of the human population, preferentially infects B cells, and establishes a lifelong infection in memory B cells ^12^. The impact of EBV in MS is controversial; some convincing recent studies indicate that infection with EBV may underlie the development of MS. Over 99% of people with MS are infected with EBV, and it has been argued that methodological differences may account for the small number of EBV-negatives ^13^. Although the effect of EBV has been extensively investigated in B cells and is also present in astrocytes and microglia of people with MS (pwMS) ^14^, the impact of EBV infection in the brain is relatively little studied. Thus, the extent and mechanism of the EBV effect remains somewhat obscure and more research is needed in this area. Numerous mechanistic links between EBV infection and MS pathology have been noted ^15^. Some of the most persuasive arguments are summarised in Table 1 ^16– 26^.

**Table 1.** Supporting and opposing arguments for EBV involvement in MS.

Supporting arguments	Opposing arguments (partly from 16)
• Epstein–Barr virus (EBV) infects and is latent in memory B cells, the same cell type shown to be critical in multiple sclerosis (MS) by successful CD20 antibody and cladribine treatment. • Genome-wide association studies (GWASs) have identified a correlation between anti-EBV nuclear antigen-1 (EBNA-1) IgG titres and MS ^27^. Human leucocyte antigen (HLA) single-nucleotide polymorphisms showed the strongest correlation with two other genes: *EVI5* and *EOMES* ^28^. • GWAS analysis reveals genetic overlap between susceptibilities to Hodgkin’s lymphoma (caused by EBV) and MS ^29^. • In B cells transformed into immortalised lymphoblasts by EBV infection, the vitamin D receptor (VDR) (nuclear receptor) interacts with the EBV nuclear protein EBNA3. This results in inhibition of VDR-promoted gene expression and provides a mechanistic link between vitamin D, EBV and MS ^30^. • EBV uses HLA DRB1 and HLA DRQ allelic variants as entry co-receptors. This is widely assumed to have immune consequences. A link between HLA variants and infectivity by EBV has not yet been made, although this has been proposed for rheumatoid arthritis ^31^. • Infectious mononucleosis is caused by EBV infection and is associated with an increased risk of MS ^32^. • EBV is found to activate the human endogenous retroviruses HERV-W/MSRV/ Syncytin-1 (human endogenous retrovirus type W/multiple sclerosis–associated retrovirus/Syncytin-1) in blood and brain cells taken from people with MS (pwMS). MSRV specifically has been reported to correlate with progression to MS ^33^ and with infectious mononucleosis. • A virus closely related to EBV, the γ1-herpesvirus CalHV3, infects marmosets, is resident in memory B cells, and is implicated in the marmoset experimental autoimmune encephalomyelitis model of experimental MS ^34^.	• Seropositivity for pwMS infected with EBV does not reach 100%, especially for children with early- onset MS ^35^. The high background of people infected with EBV clouds the interpretation. • Serum EBNA-1 antibodies are associated with MS, but no target has been found, and antibody levels are not associated with disease activity ^36^. • EBV seroconversion may just be a hygiene marker, and other parasites could be responsible for the development of MS ^37^.

Other infections such as human herpes virus 6 ^16^ and pinworms ^17^ have also been implicated in MS.

The developing picture for MS is of a disease caused by the interaction of multiple risk factors ( Figure 1), one being EBV infection. EBV could initiate changes in infected cells that lead to immune activation and a pro-inflammatory state. This alone is not sufficient to cause MS, but genetic and environmental factors then can be activated, interact and trigger the disease. Treatments defined as disease-modifying therapies (DMTs) are usually immune-modulators, other drugs treat symptoms such as limb spasticity, bladder problems, or pain. There are as yet no therapies approved to treat neurodegeneration ^18^. New immunomodulators have transformed the treatment of RRMS; at least 16 drugs are now licensed by the US Food and Drug Administration (FDA) ( https://www.nationalmssociety.org/Treating-MS/Medications) and these have generated a $20 billion market ^19^. Although the number and severity of relapses have been reduced, neurological damage still occurs from onset and the disease often continues to the progressive form. Furthermore, those patients with diagnosed PPMS have historically had no DMTs available to them.

Notably, the first drug indicated for PPMS, the anti-CD20 antibody ocrelizumab (Ocrevus ^®^), was approved by the FDA in 2017 ^20^. Ocrelizumab and other anti-CD20 therapies rapidly deplete circulating B cells. Their effectiveness in patients with PPMS and RRMS points to an important role for B cells in multiple MS forms. In a phase 3 trial, ocrelizumab was associated with lessened disease activity and progression in patients with RRMS compared with interferon beta-1a ^21^. Along the same lines, a 2018 study compared rituximab (an anti-CD20 antibody approved for other conditions) as an initial treatment for patients with RRMS and found its clinical efficacy to be notably superior to that of several other first-line DMTs, including dimethyl fumarate, natalizumab and fingolimod ^22^.

These agents have been paradigm-changing in providing the first DMT for patients with PPMS, a potentially more effective first line of treatment for RRMS, as well as turning the therapeutic focus from T cells (previously thought of as the main autoimmune effector cell in MS) to B cells. The remarkable clinical evidence with these drugs clearly illustrates that B cells play a central role in MS pathogenesis. Although this is exciting, there does remain a critical need for improvements to existing treatments in RRMS and new stand-alone treatments for SPMS and PPMS that serve to minimise adverse effects and the burden of high expense.

This review focusses on small-molecule approaches to immune-related MS therapy, covering agents on the market, in clinical trials, and some new research approaches. We specifically emphasise potential B cell–targeted therapies and the effects of already-available therapies on the B-cell population because of the recent notable success of ocrelizumab and other anti-CD20 antibodies.

Small molecules have unique and beneficial properties as therapies. The advantages include the ability to access intracellular targets and penetrate the central nervous system (CNS), low cost of manufacture, ease of administration, and the option to withdraw therapy rapidly. The primary disadvantage of small molecules is their potential lack of cellular specificity. The low cost of manufacture of small molecules compared with biological or cell-based therapies is often overlooked, but with increasing healthcare demands, a path to sustainable and affordable treatments is required for the future.

## Identifying the genes and proteins key to multiple sclerosis pathophysiology as potential drug targets

Large GWASs have identified multiple genes associated with MS (reviewed in 4). Of note, HLA-DRB1*15:01 has an odds ratio (OR) of about 3.5; OR is the ratio of people with a proposed disease-specific allele to those who do not have the disease, where 1 is no association and more than 1 indicates an increased risk. In total, 31 HLA genes have been found ^23^. Non-HLA genes have also been indicated to contribute to genetic susceptibility; in particular, a very recent communication by the International Multiple Sclerosis Genetics Consortium (IMSGC) ( Supplementary Table 1, Table 1) lists over 200 genes, showing the non-HLA-related genes ^23^.

The study builds on an earlier publication by the IMSGC detailing 97 non-HLA genes ^24^. The later gene set does not cover all of the earlier genes. For this review, we used the UniprotKB curated database to provide high-quality information on the gene sets ( Supplementary Table, Table 1) and use this information to relate the genes to specific cell types.

### B cells

In the 2018 IMSGC set, the UniprotKB database indicates that 31 genes either are highly expressed in or have some B-cell function noted ( Supplementary Table, Table 2). Only two of these genes appear to have been drugged: *CTLA4* (ipilimumab) and the cannabinoid receptor 2, *CNR2* (cannabidiol).

### Epstein–Barr virus

In the IMSGC gene set from 2013, four genes ( *PXT1*, *ZMIZ1*, *EOMES* and *TRAF3*) are linked to EBV. Further investigations have identified 47 EBV genes from transcriptomes of B cells and EBV cells infected at Latency III (LCLs) associated with MS ^25^. This study highlights the EBV-encoded cell surface protein LMP1 and its signalling pathway as a potential target for MS.

### Vitamin D

Two well-recognised MS susceptibility genes— *CYP27B1* and *CYP24A1*—control metabolism of vitamin D (1,25-dihydroxyvitamin D3) for its receptor (VDR) ^3^. Lack of 1,25-dihydroxyvitamin D3 is associated with several immune diseases. The synthesis of 1,25-dihydroxyvitamin D3 is enhanced by sunlight and thus provides a mechanistic basis for the observed latitude dependence of MS. Further studies highlighted *ZMIZ1*, *ZMIZ1*- *AS1* (AS stands for anti-sense, signifying that the single-nucleotide polymorphism is on the anti-sense strand) and *IRF8*. The VDR influences the expression and function of many other genes. *TAGAP* (T-cell activation RhoGTPase activating protein, which has a role in Th17 differentiation) and *IL2RA* were also identified by the GWASs and as 1,25-dihydroxyvitamin D3 target genes in a study on CD4 ^+^ T cells ^26^.

In the 2017 IMSGC report on the 200-plus gene set, the authors acknowledge that CNS genes may be under-represented. They partly address this by conducting an RNA-Seq study on cortex material to provide a data set more representative of CNS genes altered by the disease pathology. Only two of the RNA-Seq genes are represented in the 2013 IMSGC GWAS set: *GALC* and *RGS14*. In the 2017 IMSGC set, *RGS14* and *PVR* are common genes.

### Astrocytes

Five genes in the GWAS sets are expressed in or linked to astrocyte function ( *CLEC16A*, *IL22RA2*, *TNFRSF1A*, *CYP24A1* and *PHGDH*). Also, seven genes from these sets have a link to neurodegeneration excluding that of MS ( *GALC*, *PITPNM2*, *DIKKL1*, *SLC2A4RG*, *FCRL1* and *PHDGH*). One other gene links to neurodegeneration ( *NPEPPS*); this gene codes for an aminopeptidase which may regulate neuropeptide activity and tau levels ^38, 39^.

### Oligodendrocytes

Three genes— *OLIG3* (oligodendrocyte transcription factor), *ZNF365* (DISC1-binding zinc finger protein) and *BCAS*—are key to oligodendrocyte function. *BCAS* was recently reported as being present in MS lesions ^40^; mice lacking this gene display hypomyelination ^41^. The interaction database STRING links *BCAS1* to *QKI*, a gene thought to play a major role in oligodendrocyte differentiation and myelination ^42^.

### Intracellular organelles

***Mitochondria.*** Mitochondrial dysfunction is thought to play a role in neurodegeneration ^43^. In the GWAS and RNA-Seq studies, 23 genes, including the *CYP24A1* vitamin D susceptibility gene, link to mitochondria. Several others are linked to oxidation reduction and the electron transport chain: *COXM1*, *WWOX*, *PRDX5*, *IPYR2*, *CYB* and *ALDH2* ( Supplementary Table, Table 6). Previous studies highlight the electron transport chain as a dysregulated pathway in MS lesions, although the differently expressed genes listed in these studies ( Supplementary Table, Table 7) differ from those reported in the GWAS studies. Only two genes— *CYTB* (cytochrome b [complex III subunit 3, mitochondrially encoded]) and *CASQ1* (calsequestrin-1 [calmitine])—were reported by both the IMSG RNA-Seq 2017 study and the 2012 study. Cytochrome b is, of course, intimately involved in the generation of the proton gradient that ultimately produces ATP. Defects in the electron transport chain are also linked to reactive oxygen species (ROS) generation. Calsequestrin-1 is a calcium binding protein in muscle. Deletion of the gene in mice or mutations in the human gene or both cause muscle atrophy and mitochondrial dysfunction ^44^. Defects in this pathway underscore the rationale for anti-oxidant approaches, such as Nrf-2 activation (dimethylfumarate) ^45^ or permeability pore inhibition via mitochondrial cyclophilin D ^46^.

The picture is broadly reflective of MS pathology. However, very few of these genes have been pursued as drug targets and even fewer taken into clinical trials. A recent study attempted to concatenate all of the GWAS data and has produced two more restricted gene lists for pwMS versus control and pwMS undergoing treatment. This study identified interferon-gamma signalling as the major pathway involved ^47^. Importantly, the multiple genes associated with B-cell function/dysfunction and EBV support a role in MS pathology.

## Immune cells: small-molecule drugs

In Table 2, a method of assessing the likely CNS penetration of a compound is given where there are no published data. The scoring method used is the multiparameter optimisation method ^48^.

**Table 2.** Multiple sclerosis (MS)-relevant small molecules with activity against immune cells.

Name	Structure	Target/Mode of action	Relevance to MS	Central nervous system penetration
Bortezomib	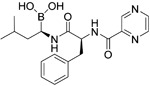	Proteasome	No studies reported	Does not cross the blood–brain barrier (BBB) ^68^ ^a^Multiparameter optimisation (MPO) = 2.8
Carfilzomib	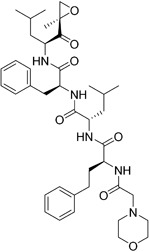	Proteasome	No studies reported	Does not cross the BBB ^69^ MPO = 1.0
Ibrutinib	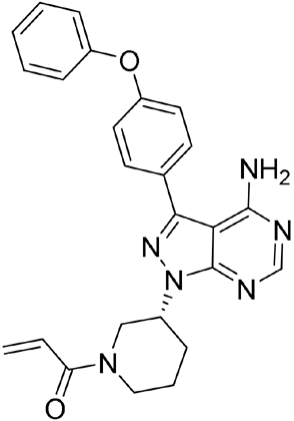	B-cell progenitor (Bruton) tyrosine kinase is highly expressed in immature B cells, monocytes and natural killer (NK) cells but not T cells.	No development reported	In mice, Ibrutinib rapidly crosses the BBB (in 0.29 hours). Brain-to- plasma ratio average for ibrutinib was found to reach 0.7 ^70^. MPO = 2.5
Evobrutinib	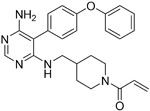	B-cell progenitor (Bruton) tyrosine kinase	Positive phase IIB results were reported in MS (7 March 2018).	Capability to pass the BBB has not been assessed. MPO = 2.0
PRN2246 Possible structure shown	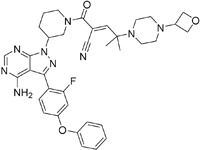	B-cell progenitor (Bruton) tyrosine kinase	Sanofi is developing a Principia MS candidate ^71^.	A recent press release confirmed that PRN2246 crosses the BBB in humans and achieves therapeutic levels in the cerebral spinal fluid ^72^. MPO = 3.4
Idelalisib	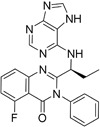	PI3Kδ is widely expressed in immune cells.	No development reported	Does not cross a healthy BBB ^73^ MPO = 2.9
CP-25 paeoniflorin-6′- O-benzene sulfonate	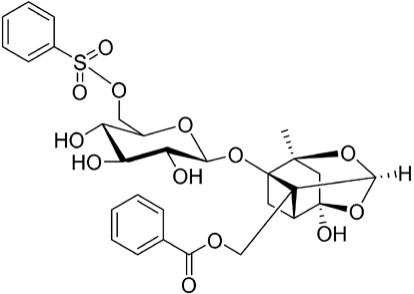	BAFF	No development reported	A recent tissue distribution and excretion study in rats suggests that CP-25 can pass through the BBB with significant differences seen between rat genders ^74^. MPO = 2.0
Cladribine	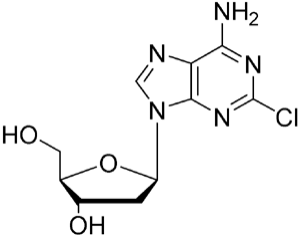	Adenosine deaminase	Positive results in phase III CLARITY study ^75– 77^ Positive results in phase III CLARITY extension study ^2^ Positive results in phase III ORACLE MS study ^78^, ^79^ Positive results in phase II ONWARD study adding oral cladribine to interferon beta-1a treatment ^80^ Long-term follow-up of safety currently active (PREMIERE) US Food and Drug Administration (FDA) accepted New Drug Application resubmission in July 2018 (Melao, FDA to Review EMD Serono’s New Request for Approval of Cladribine for Treating Relapsing MS, 2018 https:// multiplesclerosisnewstoday.com/2018/07/31/ fda-accepts-resubmitted-nda-cladribine-tablets- treatment-relapsing-ms/">https://multiplesclerosisnewstoday.com/2018/07/31/fda-accepts-resubmitted-nda-cladribine-tablets-treatment-relapsing-ms/).	Cladribine concentration in the cerebrospinal fluid (CSF) is about 25% of the plasma concentration at dose rates between 0.17 and 2.5 mg/m ^2^ per hour intravenously in patients without known meningeal disease ^54^. MPO = 3.3
Maribavir	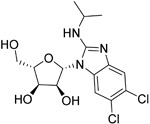	Potential Epstein–Barr virus (EBV) treatment	No studies reported	Maribavir does not cross the BBB in rats. In monkeys, maribavir levels in the brain, CSF and vitreous humour range from 4-20%, 1-2% and <1% of corresponding plasma concentration, respectively ^81^. MPO = 3.4
KAY-2-41	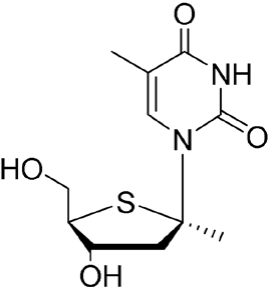	DNA polymerase in EBV-infected cells	No studies reported	Capability to pass the BBB has not been assessed. MPO = 4.2
KAY-39-149	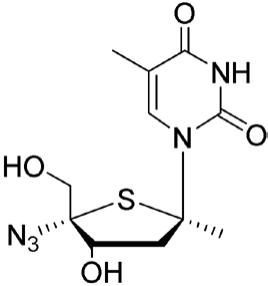	DNA polymerase in EBV-infected cells	No studies reported	Capability to pass the BBB has not been assessed. MPO = 3.2
HPMP-5-azaC	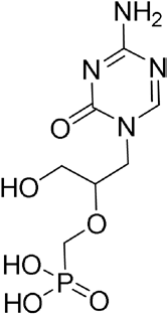	DNA polymerase	No studies reported	Capability to pass the BBB has not been assessed. MPO = 3.0
Glatiramer acetate (random peptides of the following amino acids *L-Glutamic Acid* *peptide with L-Alanine* *L-Lysine L-Tyrosine* *Acetate Salt*)		Alters antigen-presenting cell (APC) function, potentially B cells	FDA approved in 1997 as Copaxone for relapsing-remitting MS (RRMS) and clinically isolated syndrome (CIS). Two generic forms were approved in 2017.	Owing to its high polarity and hydrophilic nature, glatiramer acetate does not penetrate the BBB ^82^.
Dimethyl fumarate ( *Tecfidera*)	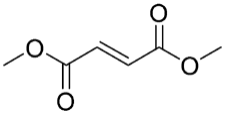	Antigen-presenting and cytokine- producing functions of B cells	FDA approved in 2013 for RRMS	Metabolite monomethyl fumarate (MMF), the primary metabolite observed after oral dimethylfumarate (DMF) dosing, crossed the BBB. MMF exposure was measured 30 min after a single oral dose of DMF (100 mg/kg) in wild- type mice. The highest concentrations were in plasma (307.2–54.79 µM), and exposures of about 100 µM were observed between peripheral organs and brain ^83^. MPO = 5.0
Fostamatinib	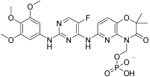	Protein tyrosine kinase syk (spleen tyrosine kinase)	No development reported	Capability to pass the BBB has not been assessed. MPO = 2.4
Fingolimod Requires phosphorylation for activation	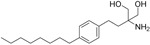	Sphingosine-1-phosphate receptor (S1P _1/3/4/5_)	FDA approved in 2011 for RRMS Approved for children and adolescents with RRMS in 2018. Phase IV study comparing fingolimod with dimethyl fumarate is currently recruiting.	FTY720 has been shown to cross the BBB in rats, dogs and humans. Tissue-to-blood partition coefficient values in rats were 22.9 for lymph nodes, 27.1 for brain, and 15.7 for thymus. MPO = 2.8
Amiselimod	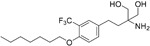	S1P _1_	Positive results in phase II MOMENTUM study for RRMS ^84^	Capability to pass the BBB has not been assessed. MPO = 2.8
Siponimod	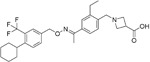	S1P _1_/ S1P _5_	Positive results in phase II trial for RRMS ^84^ Mechanistic phase III trial completed Efficacy and safety phase III EXPAND trial is currently active for patients with secondary progressive MS (SPMS).	Brain distribution was examined using an iodine-labelled analogue of siponimod, [ ^123^I]MS565. Intravenous injections gave concentrations in the rhesus monkey brain of 0.008 to 0.014 %ID/mL at around 24 hours ^85^. MPO = 2.3
Ozanimod	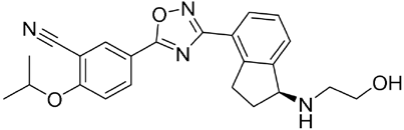	S1P _1_/ S1P _5_	Positive results in phase II RADIANCE study ^86^ Positive results in phase III SUNBEAM study ^87^	Ozanimod effectively crosses the BBB and demonstrated a high volume of distribution in both mouse and rat species, 10 and 13 L/kg, with a brain-to-blood ratio of 10:1 and 16:1, respectively ^88^. MPO = 3.7
Ceralifimod	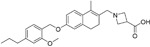	S1P _1_/ S1P _5_	Positive results in phase II and phase II extension for RRMS (DreaMS) ^89^ Merck is not pursuing phase III development.	Capability to pass the BBB has not been assessed. MPO = 3.0
Ponesimod	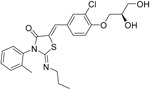	S1P _1_	Positive results in phase II ^90^ Phase II extension is active. Phase III POINT study is currently recruiting.	Capability to pass the BBB has not been assessed. MPO = 1.9
GSK2018682	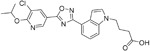	S1P _1_/ S1P _5_	Phase I trial completed	Capability to pass the BBB has not been assessed. MPO = 3.1
Laquinimod	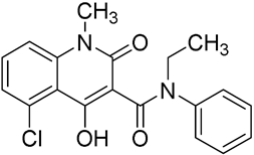	APC/nuclear factor-kappa B (NF-κB) pathway /Inhibits cytokines and lymphocyte migration into the central nervous system	Phase III trial completed for RRMS	The laquinimod concentration in the cerebrum, cerebellum and spinal cord reached about 7% to 8% in relation to the blood concentration at the time point of maximum concentration (2 hours post-dose) in healthy rats with intact BBB ^91^. MPO = 5.3
Teriflunomide	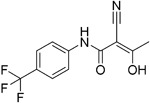	Pyrimidine synthesis inhibitor	FDA approved in 2012 for RRMS Cochrane review, only 14 mg/day delays disability ^92^ Positive results in phase III TOWER study for RRMS ^93^ Active phase III study for patients with first clinical episode was suggestive of MS (TERICIS).	Only limited penetration across the BBB (brain-to-blood ratio ≤2%). MPO = 5.4
Leflunomide	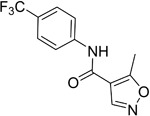	Pyrimidine synthesis inhibitor	No trials reported	MPO = 5.7 Metabolised to teriflunomide as detailed above
Mitoxantrone	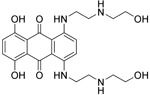	Topoisomerase II inhibitor/Induces B-cell death	FDA approved in 2000 for SPMS, progressive- relapsing MS (PRMS) and worsening RRMS but not primary progressive MS (PPMS)	Does not cross the BBB but may penetrate brain tumours ^94^ MPO = 3.3
Tofacitinib	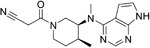	Inhibitor of the enzyme Janus kinase 1 (JAK1) and Janus kinase 3 (JAK 3)	No development reported	Shows limited distribution across the BBB with a cerebral-to-systemic blood ratio of 0.05, as assessed by either peak plasma concentration (C _max_) or area under the curve ^95^. MPO = 4.8
Mycophenolic acid	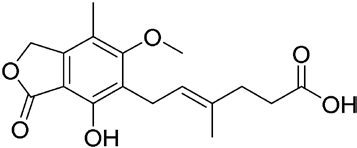	IMPDH2	Clinical trial completed ^61^	Capability to pass the BBB has not been assessed. MPO = 5.3

^a^MPO is assessed using the published method.

## Adenosine deaminase: clues to the mode of action of cladribine (Mavenclad)

**Figure 2.**
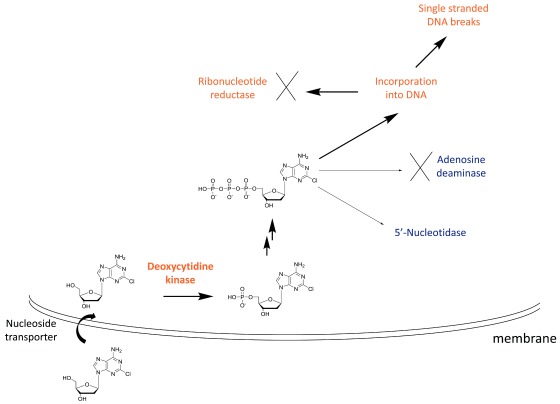
Mechanism of action of cladribine. Cladribine is taken up into cells by nucleoside transporters and then is phosphorylated to the mono-phosphate (the rate-limiting step) by deoxycytidine kinase, highly expressed in lymphocytes. Subsequent phosphorylation steps produce the active species, the triphosphate. The triphosphate cannot be efficiently degraded by adenosine deaminase, and 5′-nucleotidase has low expression in lymphocytes. This leads to high levels of the cladribine triphosphate, which is toxic to cells by a number of mechanisms, including incorporation into DNA leading to single-stranded breaks.

Adenosine deaminase is often highlighted in discussions on the mechanism of action of cladribine as the active metabolite of the drug is unable to be broken down by this enzyme ^49^. Children with adenosine deaminase deficiency have severe combined immunodeficiency disease characterised by reduced B and T lymphocyte counts ^50^. The lack of adenosine deaminase leads to an increase in dNTP levels ( Figure 2), which can lead to cytotoxicity by a number of different mechanisms. dNTPs are selectively toxic towards T and B cells ^51^ and this susceptibility has been rationalised by examining the levels of the enzymes involved in maintaining NTP levels. Lymphocytes contain low levels of the NTP catabolic enzyme 5′-nucleotidase which favours high NTP levels in the absence of adenosine deaminase ^52^.

In the case of cladribine action, the drug enters the cell via nucleoside transporters and is successively phosphorylated to the triphosphate ( Figure 2). The rate-limiting step for this process is the initial phosphorylation by cytidine kinase, an enzyme that is highly expressed in lymphocytes ( Figure 2) ^53^. Owing to the inability of cladribine triphosphate to be broken down by adenosine deaminase and the low levels of 5′ nucleotidase in lymphocytes, this active metabolite incorporates into DNA, leading to single-stranded DNA breaks and ribonucleotide reductase inhibition. This is thought to be the major cause of lymphocyte toxicity. Other mechanisms include disruption of DNA repair and an epigenetic effect.

Cladribine has medium oral absorption (37%–51% bioavailable) with a half-life of 5.7 to 19.7 hours ^54^. The drug achieves very good levels in cerebrospinal fluid (25% of plasma levels) and is metabolised mostly in the blood (in contrast to most drugs) ^55^. The testing and registration of cladribine for MS have a long and tortuous history ^56^ culminating in the CLARITY (Cladribine Tablets Treating Multiple Sclerosis Orally) phase 3 ^57^ and CLARITY extension ^2^ study. The drug is efficacious in reducing relapses following short courses of treatment (4–5 days) at 3.5 or 5.25 mg/kg with reductions in relapse rate of 57.6% and 54.5% versus placebo, respectively. Side effects such as lymphopenia and infection were predictable in line with an agent that depletes B and T cells. Cladribine is licensed for hairy cell leukaemia and B-cell chronic lymphocytic leukaemia. In 2017, it was approved for MS in Europe ^58^ and is marketed by Merck KGaA (EMD Serono in the US) as Mavenclad. Merck filed for FDA approval on 30 July 2018.

## Inosine-5′-monophosphate dehydrogenases (IMPDH1 and IMPDH2)

IMPDH1 and IMPDH2 catalyse the conversion of inosine 5-phosphate to xanthosine 5-phosphate. IMPDH2 is the rate-limiting enzyme for guanosine biosynthesis ^59^, and there are similarities with the mechanism of cladribine above. Inhibitors of these enzymes are generally immune-suppressive. Mycophenolate mofetil is an old drug which has recently shown some clinical benefit in MS ^60, 61^. It is a non-selective inhibitor of IMPDH1 and 2 and has Ki values of 40 and 10 nM, respectively ^62^. The mechanism of immune suppression has been characterised in systemic lupus erythematosus ^63^ as slowing B-cell proliferation and plasmablast formation. The lack of selectivity for mycophenolate and other older drugs has led to a search for more selective inhibitors. Sanglifehrin is a natural product with immune-suppressive properties that only recently have been characterised as working through inhibition of IMPDH2 ^64^. The spiroketal moiety of sanglifehrin is responsible for this activity, forming a ternary complex between IMPDH2, sanglifehrin, and cyclophilin A. Sappanone is a covalent and selective inhibitor of IMPDH2 over IMPDH1, binding to the Cys140 on the protein ^65^, and this type of compound may offer promise for an effective treatment with fewer side effects.

## Dihydroorotate dehydrogenase inhibitors: teriflunomide

Dihydroorotate dehydrogenase (DHODH) is a critical mitochondrial enzyme involved in the *de novo* biosynthesis of pyrimidines. Inhibiting this enzyme limits the available pyrimidine reserve needed for the increased proliferation of T and B lymphocytes seen in patients with MS ^66, 67^. This lowers the inflammatory response to auto-antigens by decreasing the number of activated T and B cells available to cross the blood–brain barrier into the CNS.

Teriflunomide acts primarily as a non-competitive and selective inhibitor of DHODH and is approved for the treatment of relapsing forms of MS (RRMS). The registration and approval of teriflunomide were due to the success of three randomised placebo-controlled trials in relapsing MS, which demonstrated that daily oral dosing of 7 to 14 mg is effective versus placebo in three key measures: relapses, disability progression and brain lesions. Clinical trial III (Oral teriflunomide for patients with a first clinical episode suggestive of multiple sclerosis, or TOPIC) showed that 72% of patients remained relapse-free on teriflunomide 14 mg versus 62% with placebo ^96^. A recent study on the effect of teriflunomide on different immune cell subpopulations in patients with MS indicated that, while teriflunomide significantly reduces absolute counts of total CD19 ^+^ B cells and mature and regulatory B-cell subsets, it affects T-cell numbers to a noticeably lesser extent and shows no detectable effect on natural killer (NK) cell numbers ^97^. However, the reduction in memory B cells is modest, and a more recent study suggests that when teriflunomide is used as a second-line treatment, relapse rates of patients with RRMS were increased compared with those who switched to dimethyl fumarate ^98^. Oral bioavailability of teriflunomide is about 100%, and peak plasma levels are achieved within 1–2 hours of ingestion ^99^. Teriflunomide is the active metabolite of leflunomide ( Table 2) ^100, 101^. Further data on teriflunomide were presented at the 2018 European Committee for Treatment and Research in Multiple Sclerosis (ECTRIMS) meeting to indicate a reduction in T-cell receptor (TCR) repertoire diversity in patients with RRMS. Metabolic analysis of T cells of patients showed increased metabolic potential over controls; the authors consider that teriflunomide can improve energy production in T cells via dihydroorotate inhibition ^102^.

## Epstein–Barr virus as a target

Should EBV be established as the trigger for MS or as a contributor to the pathology, then specific EBV anti-virals might also offer a route to treatment. A small study using autologous EBV-specific T-cell therapy has been reported ^103^ noting promising clinical improvement. Unfortunately, however, the state of EBV anti-viral chemotherapy is poor ^104^.

Maribavir, a nucleoside anti-viral, has been investigated in detail for inhibition of EBV replication ^105^. This drug initially stalled in development for cytomegalovirus (CMV) but achieved success in the treatment of haemopoietic stem cell transplant and solid organ transplant patients ^106^. It has recently been granted breakthrough status by the FDA. It is, however, not currently being developed for EBV ^107^. Maribavir works through the EBV protein kinase BGLF4 ^105^ rather than through a standard nucleoside target. Phosphonate anti-viral nucleosides, including HPMP-5-azaC, have shown EBV efficacy *in vitro* and in animal models ^108^. Herpes viruses may incorporate their own viral thymidine kinase and this is responsible for the high selectivity and low toxicity of agents such as acyclovir. Although viral EBV thymidine kinase has restricted specificity compared with other herpes virus thymidine kinases ^109^, some nucleosides such as KAY-2-41 and KAY-39-149 show high activity against EBV ^110^. No development of these agents is reported. The similarity between the mode of action of cladribine and that of anti-viral nucleosides is obvious, but there is currently no report of the activity (if any) of cladribine against EBV-infected cells. The development of EBV vaccines is problematic ^111^, but efforts are continuing and some recent success was observed in animals ^112^.

## Proteasome inhibitors bortezomib and carfilzomib

Bortezomib is a proteasome inhibitor used for the treatment of multiple myeloma and mantle cell lymphoma, two B cell–associated cancers. Bortezomib preferentially affects plasma cell differentiation and survival through its action on the nuclear factor-kappa B (NF-κB) pathway. This pathway is also key in inflammatory and autoimmune diseases ^113^. The proteasome is also directly involved with processing of MHC class I peptides ^114^. Following B-cell activation, B cells become more susceptible to proteasome inhibition ^115^. Thus, bortezomib has some selectivity in inducing apoptosis in activated B cells (plasma cells). Bortezomib has been trialled in the autoimmune diseases refractory systemic lupus erythematosus and neuromyelitis optica spectrum disorder ^116^ and demonstrated some efficacy ^117^. A negative aspect of bortezomib is the reported chemotherapy-induced peripheral neuropathy. This is caused by dysregulation of sphingosine-1-phosphate receptor-1 in astrocytes, which can be treated with fingolimod ^118^. Carfilzomib is a recently approved proteasome inhibitor for lymphomas. A recent study showed that carfilzomib together with other proteasome inhibitors had an effect on activated B cells and on naïve and—importantly from an MS perspective—memory B cells ^119^. There appears to be potential for the use of proteasome inhibitors in MS, particularly if their action on different subtypes of B cells becomes better understood and if the side effects are minimised.

## Bruton’s (B-cell) tyrosine kinase inhibitors

Bruton’s (B-cell) tyrosine kinase (BTK) is an essential kinase for the maturation of B cells together with phosphatidylinositol 3-kinase isoform p110delta (PI3Kδ). This pathway is important for autoimmune diseases and B-cell malignancies. In MS, BTK inhibitors show potential for highly specific removal of B cells and potential to diminish autoantibody release in rheumatoid arthritis models ^120^. The first covalent BTK inhibitor to be marketed was ibrutinib, a covalent tyrosine kinase inhibitor with activity against BTK and now licensed for the B-cell malignancies mantle cell lymphoma, del17p chronic lymphocytic leukaemia ^121^, and Waldenström’s macroglobulinemia ^122^. Although no development of ibrutinib for MS has been reported, two other inhibitors have reached development: evobrutinib (Merck KGgA) has reported positive phase IIB data, and PRN2246 (Principia/Sanofi) is still at an earlier stage ^123^. Quite recently, at the 2018 Congress of the ECTRIMS, Merck presented clinical data using evobrutinib in patients with RRMS and illustrated positive results by reduction of T1 ^+^Gd lesions compared with placebo, justifying future clinical studies ^124^.

## PI3Kδ inhibitors

As indicated above, PI3Kδ inhibitors can prevent the maturation of B cells and therefore are candidates for MS therapy. In contrast to BTK, this protein is also expressed in T cells. The expression of this enzyme is not restricted to B cells and thus could exhibit off-target effects. The gene *PIK3R1*—phosphatidylinositol 3-kinase regulatory subunit alpha (PI3-kinase regulatory subunit alpha)—has been listed as a priority gene from an interaction map analysis of GWAS data ^23^. PIK3R1 is linked to a primary immunodeficiency resulting in low or absent circulating B cells ^125^. So far, there have been no reports of development of idelalisib or other PI3Kδ inhibitors for MS. A black box warning has been issued for idelalisib which may hamper its use ^126^.

## B cell–activating factor and tumour necrosis factor alpha regulators

B cell–activating factor (BAFF) is a member of the tumour necrosis factor (TNF) family of receptors ^127^ and is a key survival factor for B-cell subsets encoded by the *TNFSF13B* gene. A secreted cytokine, it is a ligand for three TNF receptors: TACI, BCMS and BAFF-R. The interaction between BAFF and BAFF-R activates the canonical NF-κB signalling pathway for B-cell survival and activation. The association of BAFF with MS has been known since the early 2000s; BAFF is upregulated in astrocytes within MS lesions ^128^. A recent study demonstrated an association of a variant of *TNFSF13B* (BAFF-var) with MS ^129^. The same study showed no involvement of BAFF-var with multiple T-cell traits, consistent with an effect on B cells and particularly on memory B cells. Biological therapies that target this cytokine are available. This gene appears to be a rare example of one that is associated with the disease by genetics and has also been drugged. The actions of this cytokine and others on B cells and on the course of the disease have not been fully elucidated. Atacicept—a fusion protein of BAFF and a proliferation-inducing ligand (APRIL)—worsens MS, an effect ascribed to an increase in memory B cells ^7^.

CP-25 is a derivative of the natural product paeoniflorin ^130^, which is able to inhibit B-cell proliferation in a similar manner to rituximab (CD20 B-cell targeting antibody) and etanercept (TNF receptor chimera) and also downregulate the expression of BAFF-R on B cells. The mechanism of these effects is not reported ^131^. It will be interesting to note the progression of this compound and ones with a similar mode of action. The effect of CP-25 on B-cell subsets was more modest than that of rituximab ^131^.

## Dimethylfumarate: Tecfidera

Dimethylfumarate (DMF) is an old drug originally used to treat psoriasis. Rapid metabolism converts DMF to monomethylfumarate, the main species in circulation. This compound can react with glutathione to trigger an anti-oxidant response. The main mechanism of action is thought to be via activation of the transcription factor Nrf2, thus inducing a protective effect on cells ^45^, although different targets of DMF have been observed ^132^. Multiple actions on the immune system in pwMS have been noted ^133^, including a potentially important decrease in CD19 ^+^CD27 ^+^ memory B cells ^134^. This may in fact be a main mechanism of action due to the dramatic effects observed with other B cell–targeted therapies.

## Janus kinase inhibitors

Janus kinases (JAKs) are downstream effectors of cytokine receptors and thus are potentially useful in MS given the importance of cytokines in driving MS. Although JAK inhibitors, such as tofacitinib, are in trials for many autoimmune diseases, MS is not currently a disease indication for inhibitors of this type ^135^. The *JAK2* gene is listed as a priority gene from the protein interaction analysis in the recent GWAS ^23^.

## Sphingosine-1-phosphate receptor inhibitors

Fingolimod is a prodrug that is activated by phosphorylation with sphingosine kinases. The phosphate form can then bind and initially activate receptors sphingosine-1-phosphate 1 (S1P1), S1P3, S1P4 and S1P5 ^136^. The action on the S1P1 receptor is thought to mediate as a functional antagonism with removal of the receptor from the membrane and proteasomal degradation. The S1P1 receptor is essential for lymphocyte trafficking from lymph nodes. Although the mechanism was thought to be primarily through T cells, studies also point to an effect on astrocytes and microglia ^137^ and this may be via the S1P3 receptor ^138^. Despite increasing serum levels of BAFF in pwMS, fingolimod did not activate memory B cells or plasma cells ^139^. The overall beneficial effects of fingolimod in MS are tempered by side effects ^140^ and have stimulated the development of more selective drugs such as siponimod ponesimod and ozanimod ^136^. Only detailed clinical analysis will establish the relative effectiveness of these compounds, particularly as they must now compete with the emerging B cell–directed therapies.

## Aryl hydrocarbon receptor

Laquinimod, a quinolone-3-carboxamide derivative, is an innovative oral anti-inflammatory drug developed for the treatment of RRMS, PPMS and Huntington’s disease. Preclinical studies have shown that laquinimod reduces inflammatory cell infiltrates in the CNS. Furthermore, laquinimod suppresses clinical signs in EAE models and decreases the formation of meningeal B-cell aggregates in EAE mice ^141– 143^. Three phase 3 studies evaluating the efficacy and safety of laquinimod 0.6 mg as a treatment for RRMS have been conducted. The first study (ALLEGRO, or Assessment of Oral Laquinimod in Preventing Progression in Multiple Sclerosis) showed statistically significant differences between laquinimod and placebo in reducing the risk of sustained disability progression and rate of MRI-measured brain volume loss ^144^. The second study (BRAVO) did not reach the primary endpoint of the trial but indicated a significant reduction in disability progression and brain atrophy ^145^. CONCERTO (The Efficacy and Safety and Tolerability of Laquinimod in Subjects With Relapsing Remitting Multiple Sclerosis) was the third study to be completed, and it failed to meet its primary endpoint of a difference between patients receiving laquinimod 0.6 mg/day and those receiving equivalent placebo in confirmed 3-month disease progression; however, it did demonstrate a significant effect on reducing brain volume loss and clinical relapses ^146^.

The therapeutic effect of laquinimod was recently found to be dependent on aryl hydrocarbon receptor (AhR) activation and has been shown to induce several genes downstream linked with the AhR pathway. Among these genes were *CYP1A1* and *AHRR*, showing the highest average fold change in both naïve and EAE mice ^147^. AhR activation via laquinimod alters the phenotype of antigen-presenting cells and autoreactive T and B cells, reducing the humoral response associated with MS ^143, 148^.

## Topoisomerase II inhibitors: mitoxantrone

Topoisomerase II is a nuclear enzyme that modifies the topology of DNA by catalysing the transient breaking and rejoining of the phosphodiester backbone ^149^. Topoisomerase II inhibitors block the action of these DNA enzymes, interrupting their catalytic cycle, and are thought to give rise to the presence of protein-associated double-strand breaks, which may be lethal to a cell ^150, 151^. Two forms of topoisomerase II exist, possibly products of a gene duplication event, topoisomerase II α and β. Mitoxantrone, a synthetic antineoplastic anthracenedione, was shown to target both topoisomerase II α and β and consequently interfere with DNA repair ^152^. By inhibiting DNA replication and DNA-dependent RNA synthesis, mitoxantrone makes the cell incapable of dividing, thus suppressing the proliferation of autoreactive T cells, B cells, macrophages and other antigen-presenting cells that mediate myelin degradation ^153^. Mitoxantrone was initially proven to be effective in EAE ^154^. In addition, it has proven efficacy in the treatment of worsening RRMS, SPMS and PRMS as assessed in three controlled clinical trials ^155– 157^. However, the risks of severe adverse events of mitoxantrone are similar to those seen with other anthracyclines—myelosuppression and cardiotoxicity—and it has seen decreased use because of the introduction of newer therapies such as fingolimod ^158^.

## Glatiramer acetate

Glatiramer acetate (Copaxone) is a simple but random polymer of the amino acids Ala, Glu, Lys and Tyr ^159^. Originally conceived to mimic basic myelin, the drug is an immunomodulator and shows activity in the EAE animal model. A Cochrane review concluded that it had no effect on disease progression but showed some efficacy in “relapse related clinical outcomes” ^160^. In common with effects of other agents, its effects were thought to be T cell–related, but recent studies have pointed to a correlation with B-cell activity ^161^.

## Discussion

The study of the genetics associated with MS continues to improve our understanding of its mechanism, further implicating B cells and EBV in MS pathogenesis. However, an indication of the limitation of the genetic approach and the importance of other factors is that genetic susceptibility has thus far not proven useful in diagnosis. Even individuals with a high genetic susceptibility are unlikely to develop the disease ^4^. The ideal scenario for genetic studies is that a key target that triggers the disease or is crucial in the pathology will emerge. This idea is, of course, best illustrated by the anti-cancer drug Gleevec, which targets the bcr-abl kinase. However, even with monogenic diseases, such as Huntington’s, therapies can be slow to appear ^162^. For Alzheimer’s disease, there have been several discoveries linking particular genes and proteins to the disease but this has not produced any therapies to date. Complex multifactorial diseases such as MS would seem to be unlikely candidates for a magic bullet resulting from genetic studies.

The effectiveness of the new B cell–targeted agents, such as the CD20 antibodies and cladribine, has placed B-cell therapies at the centre of MS therapy. These therapies, however, have a non-optimal therapeutic index, and investigation of alternative B-cell agents is warranted. With cheap and effective small molecules such as cladribine already available, new agents need to demonstrate significantly improved side-effect profiles. This review concentrated on the promise of new small-molecule agents as B cell–directed drugs. Clearly, agents such as the BTK inhibitor evobrutinib have already shown some potential in clinical trials ^123^.

Accumulating evidence places EBV as central to the disease, but no effective control of EBV has been achieved as yet. The only advanced therapy likely to have EBV efficacy, maribavir, has not been trialled for EBV. Maribavir is effective in human CMV therapy, and a trial in MS would be justified. Maribavir shows good CNS penetration, allowing it to access CNS cells such as astrocytes that are known to be infected with EBV. If EBV is responsible only for activation of the immune system, then EBV-specific treatment will have an effect similar to that of current immune therapies; however, if significant EBV infection affects other cells such as astrocytes, then a more pronounced effect should be evident. While debate continues on the role of EBV in MS, it will be difficult to address this question without an effective EBV therapy. B-cell therapies remove only the latent form and do not eliminate the virus elsewhere in the body. The marmoset model is perhaps superior to rodent models as a way to evaluate MS therapies; testing anti-EBV therapies would require that the CalHV3 virus show a sensitivity similar to that of EBV to the anti-viral agent.

Treatment options for patients with MS have improved enormously over the last few decades; however, current therapies are expensive and ultimately do not prevent disease progression. Small molecules represent a more affordable and sustainable class of drugs that are favourable for MS in particular because of higher blood–brain barrier penetration. Here, we have discussed small molecules that target B cells, and EBV-infected B cells, and are currently being pursued or warrant future investigation in the context of MS. However, few of the genes and pathways targeted affect MS susceptibility genes that are implicated in the mechanism of the disease. The noteworthy progress of anti-CD20 antibodies and cladribine has brought the role of B cells in MS pathology to the forefront of current research. This opens the door to numerous exciting possibilities to develop unique, affordable and easily maintainable therapies.

## Methodology

Gene and protein data were downloaded from UniprotKB, a curated high-quality database. Gene lists were inputted to https://www.uniprot.org/uploadlists/, and the data were downloaded in tab format and saved in Excel ( Supplementary Table). Selected gene data were supplemented with PubMed searches. Gene lists were taken from the following sources: Patsopoulos 2017 ^23^, Beecham 2013 ^24^ (both IMSGC), Fischer ^43^, Sevastou ^163^ (RNA-Seq mouse EAE) and EBV genes Afrasiabi 2018 ^25^.

## Supplementary material

Supplementary Table 1 - Gene lists
